# Prediction of Shooting Events in Soccer Videos Using Complete Bipartite Graphs and Players’ Spatial-Temporal Relations

**DOI:** 10.3390/s23094506

**Published:** 2023-05-05

**Authors:** Ryota Goka, Yuya Moroto, Keisuke Maeda, Takahiro Ogawa, Miki Haseyama

**Affiliations:** 1 Graduate School of Information Science and Technology, Hokkaido University, N-14, W-9, Kita-ku, Sapporo 060-0814, Hokkaido, Japan; 2 Faculty of Information Science and Technology, Hokkaido University, N-14, W-9, Kita-ku, Sapporo 060-0814, Hokkaido, Japan

**Keywords:** shoot event prediction, soccer video, graph convolutional recurrent neural network, spatio-temporal information, Bayesian neural network

## Abstract

In soccer, quantitatively evaluating the performance of players and teams is essential to improve tactical coaching and players’ decision-making abilities. To achieve this, some methods use predicted probabilities of shoot event occurrences to quantify player performances, but conventional shoot prediction models have not performed well and have failed to consider the reliability of the event probability. This paper proposes a novel method that effectively utilizes players’ spatio-temporal relations and prediction uncertainty to predict shoot event occurrences with greater accuracy and robustness. Specifically, we represent players’ relations as a complete bipartite graph, which effectively incorporates soccer domain knowledge, and capture latent features by applying a graph convolutional recurrent neural network (GCRNN) to the constructed graph. Our model utilizes a Bayesian neural network to predict the probability of shoot event occurrence, considering spatio-temporal relations between players and prediction uncertainty. In our experiments, we confirmed that the proposed method outperformed several other methods in terms of prediction performance, and we found that considering players’ distances significantly affects the prediction accuracy.

## 1. Introduction

Data mining and machine learning technologies have made data analytics increasingly crucial in the field of sports field [1]. Quantitative evaluation of players’ and teams’ performance is a major task for enhancing competitiveness, and several methods have been proposed for various sports such as baseball [2,3] and basketball [4,5]. In soccer, such quantitative evaluation is expected to improve tactical coaching [6,7] and help players in making decisions [8,9,10]. In particular, the importance of data analytics in soccer has increased further since the International Football Association Board (IFAB) (https://www.theifab.com/, accessed on 1 February 2023) allowed the use of electronic communication devices by the coaching staff for tactical coaching during matches in 2018 [11], making it even more crucial. However, as soccer is a low-scoring and dynamic team sport, it is challenging to determine whether individual player actions contribute to match results or not [12]. Consequently, there is a need for quantitative evaluation methods that consider the complexity of soccer analytics. Soccer analytics researchers have conventionally proposed several methods to quantify the actions of each player and the team strategy by utilizing the occurrence probability of specific events such as shots and passes [13,14,15,16,17,18,19,20,21,22,23,24,25,26,27]. Since the performance metrics based on event probability are robust in the face of soccer-specific chance, they represent the team’s performance more reliably than traditional performance metrics such as ball possession, the number of shots on goal and pass rates [13]. In particular, the probability of a shoot event occurrence is often used as a substitute for the quantitative evaluation of both offensive and defensive team play since shoot events have the potential to directly impact the scoreline [12]. The conventional methods have mainly achieved the prediction of the event occurrence probability using tracking data, which consists of time-series data showing the two-dimensional positions of players on the field. Studies have shown that prediction methods based on both video and tracking data for sports such as water polo and basketball are more accurate than those relying on only one type of data [28]. Despite these findings, traditional prediction methods in soccer have not integrated visual information obtained from video data. Thus, incorporating both video and tracking data in a new prediction method is likely to lead to more accurate predictions in soccer. Moreover, while conventional deep learning methods have achieved remarkable success in various fields, some of these methods do not consider the uncertainty in their predictions [24,25,26,27]. Consequently, the generalization ability of these methods can decrease due to overfitting, leading to decreased reliability in the prediction results and overconfidence in their accuracy. Therefore, it becomes difficult to make correct decisions when introducing unreliable prediction results into tactical coaching in soccer. Since uncertainty has several aspects, such as the lack of knowledge about the predicted event, it is challenging to estimate prediction uncertainty through statistical inferences based on stochastic models. Thus, there is a need for a method that enables the evaluation of prediction uncertainty. Based on the aforementioned issues, the problems that need to be addressed are as follows.

(1) Conventional methods solely concentrate on the positional relations among players, without incorporating any visual information from video data.
(2) Conventional methods fail to account for the reliability of event occurrence probabilities derived from deep learning-based methods.

In this paper, we introduce a novel approach that predicts shoot events by considering players’ spatial-temporal relations in soccer videos through a complete bipartite graph. The occurrence of shoot events in soccer matches is closely linked to the position and movement of the players, and modeling spatio-temporal relations can aid in predicting them.

Therefore, our method utilizes a graph to represent players’ relations in each video frame, effectively incorporating the soccer domain knowledge into the treatment of visual information obtained from video data. We employ a graph convolutional recurrent neural network (GCRNN) [29,30], which which has fused a graph convolutional network (GCN) [31,32] and a recurrent neural network (RNN) [33,34], to learn the spatial and temporal relations between players in the graph. The proposed method uses visual information obtained from the video data as nodes and constructs a complete bipartite graph based on players’ distances and team information. It is worth noting that broadcast videos, which emphasize the ball carrier and include dissolves and replay scenes, may not capture the complete spatio-temporal information of players. Therefore, we focused on unedited videos that provide a birds-eye view of the entire match, which are commonly referred to as scouting videos. Overall, our method can accurately predict shooting events by effectively incorporating the spatio-temporal relations of almost all players in soccer videos. Moreover, to address the issue of prediction uncertainty, we incorporate a Bayesian neural network (BNN) [35,36] into our method. This approach allows for the quantitative evaluation of both model- and data-dependent uncertainties by treating the neural network’s weight parameters as random variables. The proposed method enables the prediction of the probability of shoot event occurrence and also allows for the calculation of the degree of uncertainty associated with making the prediction. Note that this study is an extension of our previous work [37], in which we proposed a more accurate prediction model by incorporating a complete bipartite graph with soccer-specific domain knowledge, including players’ team information. The main contributions of this study are as follows.

(1) Our novel method for predicting shoot events in soccer videos incorporates player-specific features and spatio-temporal relations using GCRNN.
(2) We propose a method that aims to enhance the robustness of predictions by providing a measure of prediction uncertainty through the use of BNN.

## 2. Pre-Processing

**Figure 1:**
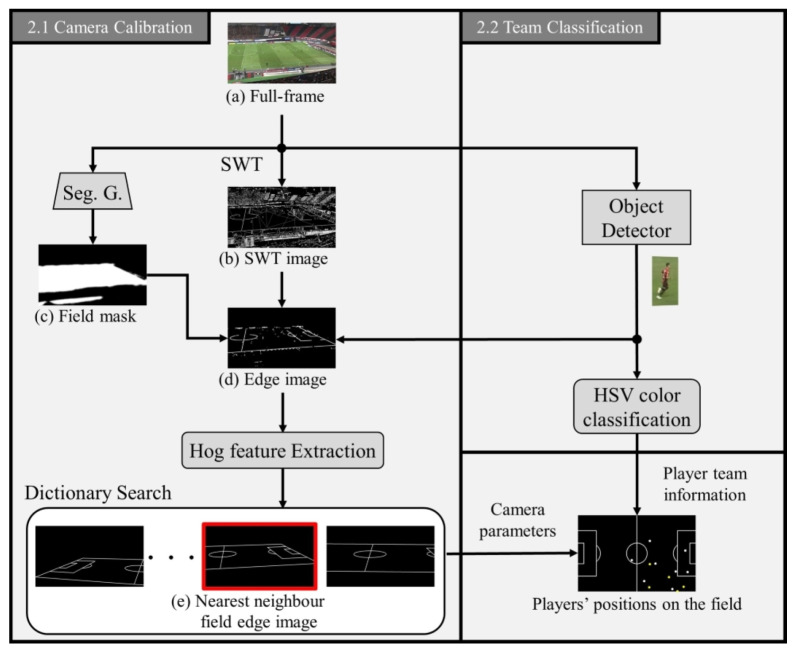
This section provides an overview of our pre-processing approach. Firstly, we use a full-frame image (**a**) as input and obtain a Stroke Width Transform (SWT) image (**b**) and a field mask (**c**) through SWT and a pix2pix-cGan segmentation generator network, respectively. This leads to an edge image (**d**) of the field. Next, we capture the corresponding camera parameters by searching for the nearest neighbor field edge image (**e**) based on HOG features. In another step, we classify the detected players into their respective teams based on HSV color. Finally, using both pieces of information, we obtain the players’ positions on the field as well as their teams.

This section describes the pre-processing methods used to construct complete bipartite graphs based on the detection of players in a soccer video. The pre-processing flow is illustrated in Figure 1. Initially, we employ a camera calibration technique to localize players on the soccer field, as explained in (Section 2.1). Furthermore, we present a technique for classifying team classification of detected players in the video (in Section 2.2).

### 2.1. Camera Calibration

To obtain the two-dimensional coordinates of the players detected in the soccer video on the field, it is necessary to estimate the camera parameters for the homography transformation. Then we use stroke width transform (SWT) [38] as an edge detector to detect the field lines on the soccer field. SWT is effective not only in preventing sparse detections but also in being robust to difficult viewpoints and noise effects such as shadows, and field stripes (both bright and dark green stripes). Therefore, it has already been confirmed to be effective in extracting the white line region on the soccer field [39]. After applying the SWT to a full-frame image (a), the resultant image (b) contains numerous edges that are irrelevant to the field lines that require removal. Consequently, we perform certain processes to eliminate the edges that are not associated with the field lines, as follows:

- Since field lines are believed to maintain a small and consistent stroke width, we apply a filter to include only strokes that are longer than 10 pixels.
- To segment strokes based on a the field color, we extract only those strokes within the 36<h<90 range in the HSV color space, as the field lines are typically found on the grass.
- We use a field mask (b) to mask off-field areas such as spectator seats and benches.
- To eliminate player regions, we use a Mask R-CNN detector [40].

The field mask (c) is obtained using the segmentation image output of the generative network from the two-conditional GAN model for detecting soccer field lines [41] with the full-frame (a) as input. The generative network of the two-conditional GAN model is based on the pix2pix network architecture [42], and it uses modules of the form convolution BatchNorm-Relu and follows U-Net [43]. Although this model is capable of generating the edge image of the field line from broadcast images, it did not work well with the scouting video frame used in this study. Therefore, we only used the segmentation image as the field mask. Finally, we can obtain an edge image (e) of the soccer field by conducting a dilation operation to remove noise.

**Figure 2:**
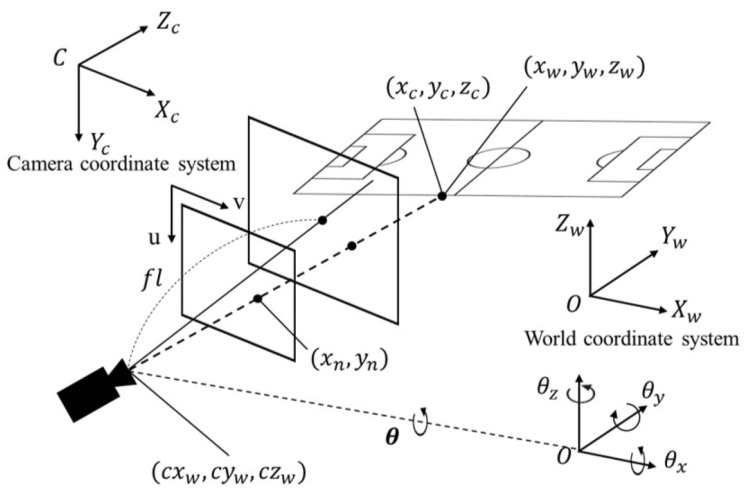
Perspective projection camera model. First, the coordinates of the field edge $\left(x_{w},y_{w},z_{w}\right)$ in the world coordinate system are transformed to their coordinates $\left(x_{c},y_{c},z_{c}\right)$ in the camera coordinate system, which originates from the camera center. Then, we obtain the projected coordinates $\left(x_{n},y_{n}\right)$ in the normalized image plane of size u×v.

As a solution for capturing camera parameters necessary for homography transformation, we propose an approach based on HOG features [44] to search for nearest neighbors in a synthetic edge image dictionary. The proposed architecture, involves finding the most similar edge image from the dictionary, giving an input edge image (e), and outputting the corresponding camera parameters. For this study, we use a perspective projection camera model (refer to Figure 2) to generate the projection-transformed image of the field template. This approach considers the virtual image plane in the pinhole camera model as the true image plane and generates images that are used as synthetic field edge images.

**Figure 3:**
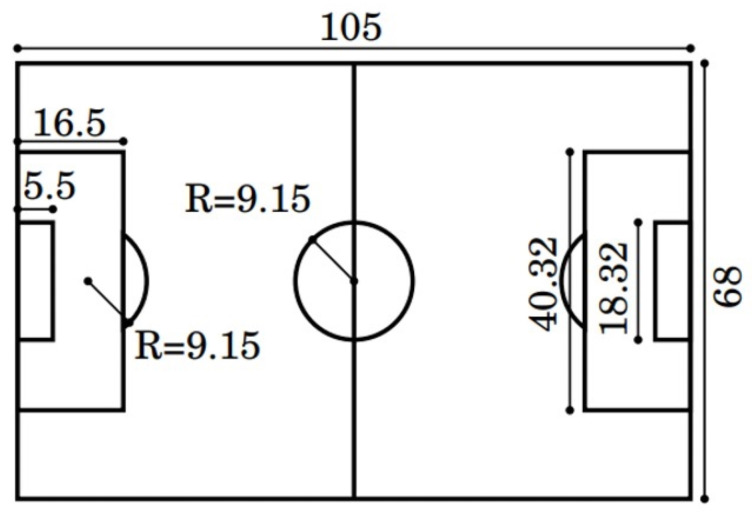
Standard soccer field dimensions. These are expressed in meters for each measurement.

For the field template, we have used the standard field (refer to Figure 3), which is commonly used in international matches like the World Cup and the Olympic Games, and is defined by the Federation Internationale de Football Association (FIFA) (https://www.fifa.com/, accessed on 1 February 2023). The equation for the projection transformation is presented below:

(1) $$\begin{array}{c} Z_{n}\left[\begin{array}{c} x_{n}\\ y_{n}\\ 1 \end{array}\right]=\left[\begin{array}{ccc} fl_{x} & 0 & u\\ 0 & fl_{y} & v\\ 0 & 0 & 1 \end{array}\right]\left[\begin{array}{c} x_{c}\\ y_{c}\\ z_{c} \end{array}\right]=\left[\begin{array}{ccc} fl_{x} & 0 & u\\ 0 & fl_{y} & v\\ 0 & 0 & 1 \end{array}\right]R\left(\theta \right)\left[\begin{array}{c} x_{w}-cx_{w}\\ y_{w}-cy_{w}\\ z_{w}-cz_{w} \end{array}\right], \end{array}$$

where ${\left[x_{w},y_{w},z_{w}\right]}^{⊤}$ and ${\left[cx_{w},cy_{w},cz_{w}\right]}^{⊤}$ represent the field edge coordinates and camera coordinates in the world coordinate system, respectively. $R\left(\theta \right)\;\left(\theta =\left[\theta_{x},\theta_{y},\theta_{z}\right]\right)$ denotes the Rodrigues rotation matrix with the camera rotation angle θ. The camera parameters obtained above are used to calculate the field edge coordinates ${\left[x_{c},y_{c},z_{c}\right]}^{⊤}$ in the camera coordinate system. The value of $\left(fl_{x},fl_{y}\right)$ and $\left(u,v\right)$ are determined based on the camera scaling and the size of the projection-transformed image, respectively. As a result of the aforementioned projection transformation, we obtain the field edge coordinates ${\left[x_{n},y_{n}\right]}^{⊤}$ in the image coordinate system, which are then used to retrieve the field edge image. These camera parameters are constrained within specific ranges based on the environmental conditions to be captured. In this study, our focus is on scouting soccer videos during a home game of a team in the Japan J1 League. Therefore, we experimentally determined the camera position to be ${\left[cx_{w},cy_{w},cz_{w}\right]}^{⊤}={\left[-7,105,36\right]}^{⊤}$. For the camera rotation angles, we limited the ranges to $\theta_{x}\in \left[-75,-65\right],\;\theta_{y}\in \left[-20,20\right]\;\mathrm{and}\;\theta_{z}\in \left[-20,20\right]$, respectively. $\left(fl_{x},fl_{y}\right)$ were restricted to the range $fl_{x}=fl_{y}\in \left[1200,2000\right]$ to ensure that at least 1/3 of the field fits within the template projection image. $\left(u,v\right)$ were set to 640 and 360, respectively. In addition, each camera parameter $\theta_{x}$, $\theta_{y}$, $\theta_{z}$, $fl_{x}$, $fl_{y}$ is varied in increments of $\delta_{\left(\theta_{x}\right)}=\delta_{\left(\theta_{y}\right)}=\delta_{\left(\theta_{z}\right)}=1$ and $\delta_{\left(fl_{x}\right)}=\delta_{\left(fl_{y}\right)}=50$, respectively, within the limited range ($\theta_{x}\in \left[-75,-65\right]$, $\theta_{y}\in \left[-20,20\right]$, $\theta_{z}\in \left[-20,20\right]$, and $fl_{x}=fl_{y}\in \left[1200,2000\right]$). Here, $\delta_{\left(\cdot \right)}$ represents the stride of each camera parameter. After computing the HOG features of the template projection images and the edge image (d), we obtain the camera parameters of the most similar template projection image. That is the optimal ones, by performing nearest neighbor search using the Euclidean distance of those features.

### 2.2. Team Classification

This subsection describes the method used to classify players into teams, which is necessary for constructing a complete bipartite graph. To detect players in each frame, we use the well-known object detection method Mask R-CNN [40]. Mask R-CNN is a single-task framework that extends Faster R-CNN [45] and is capable of performing instance segmentation and object detection and instance simultaneously. Instance segmentation is a more challenging task than semantic segmentation as it involves classifying each pixel in an image according to both the object class and the specific instance it belongs to, whereas semantic segmentation only classifies each pixel according to its object class. Thus, we can obtain the region of each player by applying Mask R-CNN, and from the pixels of those players’ region, our method identifies the team to which each player belongs using a color-based classification method [46] that is based on the HSV uniform color. To minimize the effects of unrelated people such as referees, bench players, coaches, and photographers, we only include the field players (i.e., excluding goalkeepers) of the home and away teams, respectively, in the construction of the complete bipartite graph. These players are identified from the detected persons in the video frame. Here, it is worth nothing that while referees and goalkeepers can be distinguished based on their uniform color, it may not be possible to identify substitute players and coaches using this approach. To address this issue, our method determines whether a detected person is a field player or not by utilizing the coordinates of the detected person on the field, which are obtained through camera calibration.

Thus, the field players’ coordinates on the field can be obtained by using the obtained camera parameters obtained from the previous steps and mapping the field player’s coordinates in the video frame to the field (as shown in the lower right of Figure 1).

## 3. Method for Prediction of Shoot Events

**Figure 4:**
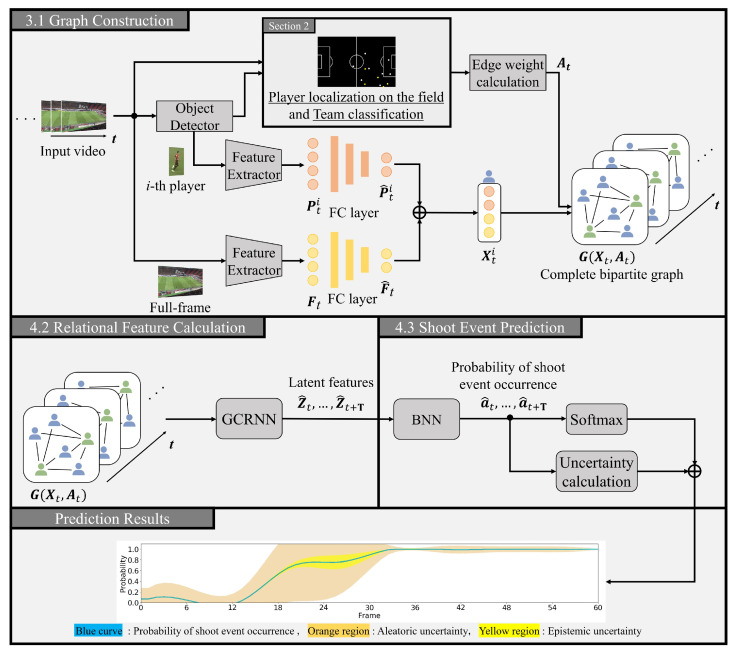
Overview of the proposed method. Our method starts by extracting visual features of each detected player and full-frame at each time step form a given soccer video input. A complete bipartite graph is constructed with these visual features as node features. We then use GCRNN to calculate latent features. Next, our method predicts the probability of shoot event occurrence based on BNN, and we obtain the resulting predictions along with their corresponding prediction uncertainties. The overall process is illustrated in Figure.

In this section, we provide a detailed explanation of the proposed method for predicting shoot events, as illustrated in Figure 4. Specifically, our approach involves constructing a complete bipartite graph using visual features of player regions and a full-frame at each time step, as described in Section 3.1. Next, we use a graph convolutional recurrent neural network (GCRNN) [29,30] to compute latent features by incorporating spatio-temporal relations between players, as described in Section 3.2. Finally, we make use of a Bayesian neural network (BNN) [35,36] to predict the probability of a shooting event occurring while considering the prediction uncertainty. This is further elaborated in Section 3.3.

### 3.1. Construction of Complete Bipartite Graphs

This subsection explains the construction process of a complete bipartite graph. To effectively incorporate visual information obtained from soccer video data along with domain knowledge, our proposed method represents the relationships between players in each video frame as a complete bipartite graph $G_{t}$. Initially, we detect players in soccer videos using the widely-used object detection and segmentation method, Mask R-CNN [40]. After detection, we classify players into teams based on their uniform colors, as described in See Section 2.2). Next, to extract the visual features of each player and the full frame at time step *t*, we feed the rectangular region of each player detected by Mask R-CNN and the full-frame at time step *t* into ResNet-101 [47], which is a pre-trained feature extractor with ImageNet [48]. The resulting features are denoted as $P_{t}$ and $F_{t}$, respectively. To enhance the feature representation capacity of the graph data, we obtain low-dimensional features ${\hat{P}}_{t}$ and ${\hat{F}}_{t}$ of each player and full-frame using a fully connected layer. Then, we concatenated these features and introduce the resulting feature vectors as a graph node $X_{t}$ according to the following formula:

(2) $$\begin{array}{c} {\hat{P}}_{t}=\mathrm{FC}\left(P_{t}\right),\;{\hat{F}}_{t}=\mathrm{FC}\left(F_{t}\right), \end{array}$$

(3) $$\begin{array}{c} X_{t}^{\left(i\right)}=\left[{\hat{P}}_{t}^{\left(i\right)},{\hat{F}}_{t}\right], \end{array}$$

where FC(·) denotes the fully connected layer, and ${\hat{P}}_{t}^{\left(i\right)}$ are i-th player’s low-dimensional visual features at time step *t*.

It has been reported that in soccer matches, other players in the target player’s action range are especially crucial to make quick and accurate decisions [49]. We represent the graph $G_{t}$ with the adjacency matrix $A_{t}$ at time step *t* using edge weights that consider players’ distances and team information, which are crucial factors in soccer analysis. The proposed method specifically introduces a complete bipartite graph $G_{t}$ where edge weights are only between players from different teams. The adjacency matrix $A_{t}$ at time step *t* is then calculated as follows:

(4) $$\begin{array}{c} A_{t}^{\left(i\;j\right)}=\left\{\begin{array}{cc} 0 & \left(\mathrm{team}(i)=\mathrm{team}(j)\right)\\ \frac{\exp\{-d\left({r_{t}}^{i},{r_{t}}^{j}\right)/w\}}{\sum_{i\;j}\exp\left\{-d\left({r_{t}}^{i},{r_{t}}^{j}\right)/w\right\}} & \left(\mathrm{team}(i)\neq \mathrm{team}(j)\right) \end{array}\right., \end{array}$$

where $r_{t}$ denotes the position on the field of each player detected by Mask R-CNN (See Section 2.1) and $d({r_{t}}^{i},{r_{t}}^{j})$ represents the Euclidean distance between *i*-th and *j*-th players. The value *w* is a constant used to balance the edge weights, and the function team (·) is used to classify the teams of the detected players (refer to Section 2.2). The proposed method incorporates the distances and team information of both players into the graph, allowing for the utilization of player relations that accurately reflect the intricate match situation.

### 3.2. Calculation of Spatio-Temporal Relational Features

**Figure 5:**
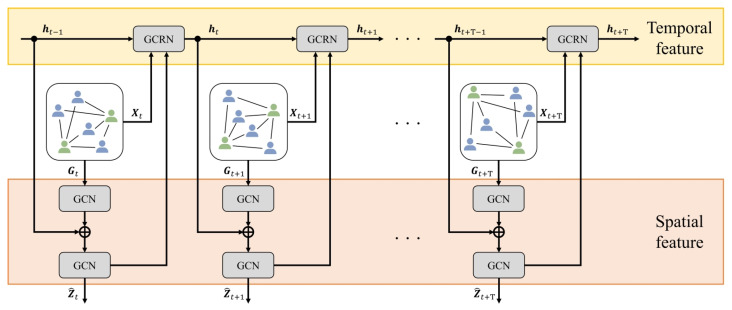
The GCRNN architecture comprises stacked GCN layers that initially learn the spatial relations. By inputting the RNN’s hidden state $h_{t-1}$ into the second GCN layer, the method can calculate the latent feature $Z_{t}$, considering both temporal and spatial relations. This process in repeated recursively to capture latent features at each time step, as illustrated in Figure.

This subsection outlines the calculation method of spatio-temporal features between players using GCRNN, which combines a graph convolutional network (GCN) [31,32] and a recurrent neural network (RNN) [33,34]. Figure 5 illustrates the architecture of GCRNN. To learn the spatial relations between players, the proposed method utilizes stacked GCN layers. Additionally, the hidden state $h_{t-1}$ of the RNN is introduced to enhance the second GCN layer, as shown bellow:

(5) $$\begin{array}{c} Z_{t}=\mathrm{GCN}\left(\left[\mathrm{GCN}\left(X_{t},A_{t}\right),h_{t-1}\right],A_{t}\right), \end{array}$$

where $X_{t}$ and $A_{t}$ represent the graph node and adjacency matrix, respectively, of the complete bipartite graph constructed in Section 3.1. The proposed method incorporates $h_{t-1}$ into the final GCN layer, enabling the calculation of the latent feature $Z_{t}$ while considering both temporal and spatial relations. Furthermore, the proposed method inputs the latent feature $Z_{t}$, which is fused with the corresponding graph node features $X_{t}$, into the RNN. The hidden state $h_{t}$ is then updated for the next time step. As the number of players in a frame of the soccer video may vary across different time steps, it is essential to consider the dynamic changes of node features in the graph. To capture temporal relations, the proposed method utilizes a Graph Convolutional Recurrent Network (GCRN) [34], which is one of the RNN models. The hidden state $h_{t}$ is updated recurrently according to the following formula:

(6) $$\begin{array}{c} h_{t}=\mathrm{GCRN}\left(\left[Z_{t},X_{t}\right],h_{t-1}\right). \end{array}$$

Through the input of $X_{t}$ and $Z_{t}$ into the GCRN, the proposed method can obtain the hidden state $h_{t}$ while taking into account both player-specific and relational features. This iterative process allows our model to capture latent features $Z_{t}$ while considering spatio-temporal relations at each time step.

### 3.3. Event Prediction with Its Uncertainty

This subsection demonstrates the prediction of shoot event occurrences with their uncertainty based on BNN. BNN allows for the estimation of both model- and data-dependent uncertainty through the inference result by treating the weights of the neural network as random variables. As a result, whereas a simple neural network model is susceptible to incorrect predictions for extrapolation due to the overfitting problem, BNN can mitigate this issue. In particular, since soccer matches may not always follow the same pattern as the training data, overfitting the training data could lead to a decrease in the model’s ability to make accurate predictions. Therefore, by incorporating BNN, the proposed method enhances the accuracy of shoot event predictions in soccer matches. With the latent features ${\hat{Z}}_{t}$ of unknown data as input, the probability of a shoot event occurrence ${\hat{a}}_{t}$ can be calculated by the predictive distribution $p({\hat{a}}_{t}|{\hat{Z}}_{t})$ using the following equation:

(7) $$\begin{array}{c} p\left({\hat{a}}_{t}|{\hat{Z}}_{t}\right)=\int p\left({\hat{a}}_{t}|{\hat{Z}}_{t},w\right)p\left(w|D\right)dw, \end{array}$$

where $p({\hat{a}}_{t}|{\hat{Z}}_{t},w)$ and p(w|D) represent the predictive distribution for a given value of network parameters w and posterior distribution of network parameters w, respectively. Network parameters w are sampled from a Gaussian distribution $p\left(w\right)∼N\left(\mu ,\sigma^{2}\right)$, where the mean μ and variance $\sigma^{2}$ are learned using the training dataset $D=(Z_{t},a_{t})$.

To compute the true posterior distribution p(w|D) of network parameters using Bayes’ theorem, it is necessary to calculate the marginal distribution ∫p(D|w)p(w)dw, in addition to the likelihood p(D|w) and the prior distribution p(w) of w. Because using a posterior distribution p(w|D) to obtain predicted probabilities is equivalent to employing an ensemble of numerous neural networks, this approach is not feasible for a neural network of a practical size. To address this issue, the proposed method utilizes Bayes-by-Backprop [36], a variational inference method that approximates the posterior distribution p(w|D) with the following equation:

(8) $$\begin{array}{c} \alpha^{*}=\underset{\alpha }{arg min}\sum_{k=1}^{K}\left[\log q(w_{k}|\alpha )-\log p(w_{k})-\log p(D|w_{k})\right], \end{array}$$

where $w_{k}$ represents the k-th Monte Carlo sample drawn from the variational posterior $q(w_{k}|\alpha )$. The variational posterior parameters $\alpha =(\mu ,\sigma^{2})$ are updated using the re-parameterization trick and gradient descent. This technique is a well-known variance reduction method in Bayesian statistics and uncertainty quantification [50]. It is important to note that each term in this approximation cost is dependent on a specific weight obtained from the variational posterior. The proposed method consider the first and second terms as loss functions, denoted as $L_{\mathrm{VPOS}}$ and $L_{\mathrm{PRI}}$, respectively. The third term refers to the negative log-likelihood, and minimizing this term results is equivalent to minimizing the mean squared error. Therefore, the proposed method uses the exponential binary cross entropy as the loss function $L_{\mathrm{EXP}}$, which is expressed as:

(9) $$\begin{array}{c} L_{EXP}=\sum_{t=1}^{T}-e^{-\max(0,\frac{T_{o}-t}{\mathrm{fps}})}\log a_{t}^{\;(p)}+\sum_{t=1}^{T}-\log\left(1-a_{t}^{\;(n)}\right), \end{array}$$

where fps represents the frame rate of the input soccer videos, and $T_{o}$ represents the time of shoot events. Here, $a_{t}={\left(a_{t}^{\left(n\right)},a_{t}^{\left(p\right)}\right)}^{⊤}$ represents the negative and positive probability of the training dataset at time step *t*, respectively, measured in units of probability.

The proposed method involves multiple forwards passes at each time step to obtain multiple prediction distribution results, which are then used to calculate a prediction distribution. The mean and variance of this distribution are considered as the expected value and the prediction uncertainty, respectively. Furthermore, the predictive uncertainty, that is, the variance, can be decomposed into the aleatoric uncertainty $U_{t}^{alt}$ and the epistemic uncertainty $U_{t}^{ept}$ [51], allowing for a better understanding of the source of the uncertainty.

(10) $$\begin{array}{c} U_{t}^{alt}=\frac{1}{M}\sum_{m=1}^{M}\left[\mathrm{diag}\left({\hat{a}}_{m}\right)-{\hat{a}}_{m}{\hat{a}}_{m}^{⊤}\right], \end{array}$$

(11) $$\begin{array}{c} U_{t}^{ept}=\frac{1}{M}\sum_{m=1}^{M}\left({\hat{a}}_{m}-\bar{a}\right){\left({\hat{a}}_{m}-\bar{a}\right)}^{⊤}, \end{array}$$

where *M* denotes the number of forward passes. ${\hat{a}}_{m}={\left({\hat{a}}_{t}^{\left(n\right)},{\hat{a}}_{t}^{\left(p\right)}\right)}_{m}^{⊤}$ represents the output value obtained from the *m*-th layer in BNN, consisting of the predicted negative probability ${\hat{a}}_{t}^{\left(n\right)}$ and positive probability ${\hat{a}}_{t}^{\left(p\right)}$ of the unknown data at time step *t*. $\bar{a}$ is the average of output ${\hat{a}}_{m}$ from each layer of the BNN. The aleatoric uncertainty $U_{t}^{alt}$ is a measure of the inherent variability in the latent features calculated by the GCRNN, which serves as input to the BNN, and is independent of the specific model used. In contrast, the epistemic uncertainty $U_{t}^{ept}$ is a measure of the model’s uncertainty about its own predictions, and decreases as the model observes more training data, and confidence in the model is expected to increase. In the proposed method, we utilize the uncertainty-guided ranking loss $L_{\mathrm{RANK}}$ [30] that leverages the epistemic uncertainty $U_{t}^{ept}$ to estimate the predictive capability of the model for the input data.

(12) $$\begin{array}{c} L_{RANK}=\max\left(0,\mathrm{trace}\left(U_{t}^{ept}-U_{t-1}^{ept}\right)\right). \end{array}$$

Finally, the prediction model is trained to minimize the coupling loss function L given by the following equation:

(13) $$\begin{array}{c} L=L_{\mathrm{EXP}}+\lambda_{1}\cdot \left(L_{\mathrm{VPOS}}-L_{\mathrm{PRI}}\right)+\lambda_{2}\cdot L_{\mathrm{RANK}}, \end{array}$$

where the constants $\lambda_{1}$ and $\lambda_{2}$ are used to balance the values of the loss functions. By utilizing BNN, the proposed method can make predictions of shoot events while accounting for uncertainty. The proposed method combines latent features that consider the detailed relations between players with uncertainty modeling to accurately predict shoot event occurrences.

## 4. Experimental Results

This section presents experimental results that verify the effectiveness of the proposed method fin predicting shoot events. Subsequently, we will describe the experimental settings (in Section 4.1), followed by a performance evaluation and discussion (in Section 4.2).

### 4.1. Experimental Settings

For this experiment, we created a dataset consisting of 400 video clips extracted from scouting soccer videos of 31 matches played during the 2019 and 2020 seasons of the Japan J1 League. Each video clip contained an attacking scene captured at 10 fps for 6 s, with each frame having a size of 1280×720 pixels. Positive samples were defined as shooting scenes, while negative samples were defined as non-shooting attacking scenes. We randomly selected 80 video clips (40 positive and 40 negative samples) for the test dataset and used the remaining 320 video clips (160 positive and 160 negative samples) for training. The time step $T_{o}$ in which the shoot event occurs in positive samples is determined manually through our observations. To determine the probability $a_{t}={\left(a_{t}^{\left(n\right)},a_{t}^{\left(p\right)}\right)}^{⊤}$ of shoot events in the training data, we set $a_{t}={\left(1,0\right)}^{⊤}$ at all time steps for negative samples. For positive samples, we set the probability as follows:

(14) $$\begin{array}{c} a_{t}^{\left(n\right)}=1-a_{t}^{\left(p\right)},\;a_{t}^{\left(p\right)}=\left\{\begin{array}{cc} 0 & \left(t<T_{o}\right)\\ 1 & \left(t\;≧\;T_{o}\right) \end{array}\right.. \end{array}$$

To balance the edge weights and loss scale values, we experimentally set *w* to 1050 in Equation (4) and $\lambda_{1}$ and $\lambda_{2}$ to 0.001 and 10, respectively, in Equation (13). The mixing ratio, which is a hyperparameter of the prior distribution, was set to 0.5. The number of forward passes, *M*, was determined by Bayes-by-Backprop [36] to be 2 during the training phase and 10 during the testing phase. In the training phase, we initialized the learning rate to $5\times 10^{-4}$ and used the ReduceLROnPlateau scheduler in conjunction with the Adam optimizer [52] to train the model with a batch size of 16 for 60 epochs. Moreover, we used the neural network with one layer as the FC layer in the graph construction for calculating ${\hat{P}}_{t}$ and ${\hat{F}}_{t}$ that had the 256 dimensions, that is, the dimension of $X_{t}$ became 512. In addition, we adopted the two GCN with one layer. The first GCN transformed graph node $X_{t}$ and adjacency matrix $A_{t}$ into the 256-dimensional feature, and the second GCN transformed the outputs of the first GCN with the hidden state $h_{t-1}$ and adjacency matrix $A_{t}$ into the latent feature $Z_{t}$. We set the dimensions of $h_{t}$ and $Z_{t}$ as 256 and 128, respectively. The GCRN in the GCRNN also had one layer that transformed the graph node $X_{t}$, latent feature $Z_{t}$ and hidden state $h_{t-1}$ into the next hidden state $h_{t}$. Finally, we input the latent feature $Z_{t}$ and calculated the prediction results by using BNN with two layers that had 64 and 2 (probabilities of positive or negative shoot event occurrence) nodes, respectively. To evaluate the efficacy of our proposed method (Ours) in predicting shoot events, we implemented several comparative methods, namely ASs1-4 and CMs1-2.

**AS1:**  This refers to our previous approach [37], which is similar to the proposed method but uses a complete graph without any team information.
**AS2:**  This method builds upon our previous method but utilizes a complete bipartite graph that does not consider the distances between players.
**AS3:**  This method is similar to our proposed method but employs a complete graph without incorporating edge weights in the graph representation.
**AS4:**  This method uses the full-frame visual features, which applies a field mask obtained in Section 2.1 to the full-frame, as node features in the proposed method.

To our knowledge, our previous study [37] is the only method that predicts shoot event occurrence using visual information. The following additional methods were adopted as comparison methods, in addition to the comparative methods mentioned earlier.

**CM1:**  This is a method for predictiong traffic accidents in dashcam videos using Dynamic-Spatial-Attention Recurrent Neural Network (DSA-RNN), as presented in the work of [53]. The DSA-RNN facilitates prediction by dynamically allocating soft-attention [54] to candidate objects in each frame, collecting cues, and learning the temporal relationships of all cues.
**CM2:**  This is a state-of-the-art method for video classification that uses the Video Vision Transformer (ViViT) [55]. ViViT tokenizes the video by dividing it into either 2-D spatial or 3-D spatio-temporal grids. The video can be classified by feeding the tokens extracted from it into the transformer encoder.

CM1 is a prediction method for traffic accidents that considers the spatio-temporal relationships between objects in the video, and thus we included it as a benchmark method for comparison in this study. Furthermore, as the classification performance of the model is indicative of its ability to accurately predict shoot events, we also used CM2 as another benchmark method for comparison.

In our experiment, we assessed the prediction performance of shoot event occurrence using Average Precision (AP) and F1-score as indicators of prediction accuracy, and mean Time to Event (mTTE) as an indicator of prediction earliness for positive samples. The AP and F1-score are calculated as follows:

(15) $$\begin{array}{c} \mathrm{AP}=\int_{0}^{1}\mathrm{Precision}(s)d\left[\mathrm{Recall}(s)\right], \end{array}$$

(16) $$\begin{array}{c} F1-\mathrm{score}\left(s\right)=\frac{2\times \mathrm{Recall}(s)\times \mathrm{Precision}(s)}{\mathrm{Precision}(s)+\mathrm{Recall}(s)}, \end{array}$$

where *s* denotes the threshold used to determine whether the model predicted a future shoot event. Based on the prediction results, Recall(s) and Precision(s) were calculated as follows:

(17) $$\begin{array}{c} \mathrm{Precision}\left(s\right)=\frac{\mathrm{TP}(s)}{\mathrm{TP}(s)+\mathrm{FP}(s)}, \end{array}$$

(18) $$\begin{array}{c} \mathrm{Recall}\left(s\right)=\frac{\mathrm{TP}(s)}{\mathrm{TP}(s)+\mathrm{FN}(s)}, \end{array}$$

where TP(s), FP(s), and FN(s) represent the numbers of videos predicted as true-positive, false-positive and false-negative at threshold *s*, respectively. Details of the mTTE calculation are provided below.

(19) $$\begin{array}{c} \mathrm{mTTE}=E[\mathrm{TTE}(s)], \end{array}$$

(20) $$\begin{array}{c} \mathrm{TTE}\left(s\right)=\max\{T_{o}-t|res\left(t\right)\geq s\}, \end{array}$$

(21) $$\begin{array}{c} res\left(t\right)=\frac{\exp\left({\hat{a}}_{t}^{\left(p\right)}\right)}{\exp\left({\hat{a}}_{t}^{\left(n\right)}\right)+\exp\left({\hat{a}}_{t}^{\left(p\right)}\right)}. \end{array}$$

### 4.2. Performance Evaluation

#### 4.2.1. Quantitative Results

The experimental results with varying video clip lengths are presented in Table 1. According to Table 1, the proposed method outperforms all other methods in terms of prediction performance for many video clip lengths. Comparing the proposed method with ASs1-3, it can be concluded that the use of player relations reflecting more detailed match conditions, and the inclusion of players’ distances and team information in the graph representation of the proposed architecture, is effective in predicting shoot event occurrence. Comparing the proposed method with AS4, it can be concluded that the inclusion of off-field visual information is also effective in predicting shoot event occurrence. The comparison of the proposed method with CM1 indicates that the proposed method is more effective in predicting shoot event occurrence than CM1, a video-based event prediction method that achieved high performance in a different task. Here, CM1 achieves high prediction accuracy, although not as high as the proposed method. CM1 adopts DSA-RNN, which applies dynamic soft attention to the features by fused frame- and object-level features of each video frame and obtaining the weighted attention level for each object. Therefore, it is suggested that CM1 was able to accurately predict shoot events by appropriately considering the relationships between players in the video. The comparison between the proposed method and CM2 indicates that the proposed method achieves higher classification accuracy than the state-of-the-art video classification method. Here, the results of CM2 were not as good as those of all other methods, and there may be two possible reasons for this problem. The first reason may be the insufficient scale of the dataset. ViViT used in CM2 is a transformer-based method, and it was reported that its generalization performance was low in the case of a small dataset due to weak inductive bias, and a large dataset was required to achieve a high-performance model [56,57]. In this experiment, the training dataset consisted of only 320 video clips, which may have contributed to the suboptimal classification results. The other reason is the potential difficulty of predicting the occurrence of the shooting event based solely on full-frame visual information. One study has reported that video-based methods alone are not as effective as location-based methods in prediction sports match events [28]. Moreover, recent research on event classification tasks in soccer matches, such as shoot, pass, and corner kicks, have demonstrated high performance by jointly leveraging not only full-frame visual information but also audio and players’ visual information [58]. Thus, it can be concluded that CM2 did not achieve good classification performance.

In the experiment, since Ours, ASs1-4, and CM1 predict the probability of a shoot event occurrence at each time step, we also evaluated the prediction accuracy at the time step $T_{o}$ when the shooting event occurs and the prediction earliness for positive samples. These experimental results are shown in Table 2. As shown in Table 2, although the proposed method exhibits the best performance in terms of AP and F1-score, it has been observed that ASs2-3 predicted positive samples correctly at an earlier stage compared to the proposed method. It should be noted that if the model predicts that most video clips are positive at an early stage, the resulting AP may not be accurately reflect the model’s performance due to overfitting on positive samples, despite obtaining a high mTTE. Predicting shoot events at an early stage may support players in making timely decisions based on the outcome. However, obtaining a high TTA without ensuring high accuracy does not hold much significance. In our model, we use the loss function $L_{\mathrm{EXP}}$ in Equation (9), where the exponential increase in loss with time for positive samples is intended to strike a balance between the earliness and accuracy of event prediction during training. Table 1 shows that the proposed method and AS1 exhibit stable AP as the video length increases, whereas ASs2-3 shows a decreasing trend in AP. Therefore, our model confirms that methods that consider the distance of players (that is, Ours and AS1) can predict the occurrence of shoot events more meticulously and precisely compared to ASs2-3. From our experiments, we indicate the effectiveness of the proposed method in the perspectives of the ablation studies and the comparison with CMs1-2. Besides, although there are some other event prediction methods, e.g., Graph and Spatio-temporal Continuity based framework (GSC) [59] that is one of the state-of-the art methods, the two contributions mentioned in Section 1 have been well demonstrated by comparing the proposed method with CMs1-2. Thus, we consider the comparisons for the other event prediction methods in the future work.

#### 4.2.2. Qualitative Results

**Figure 6:**
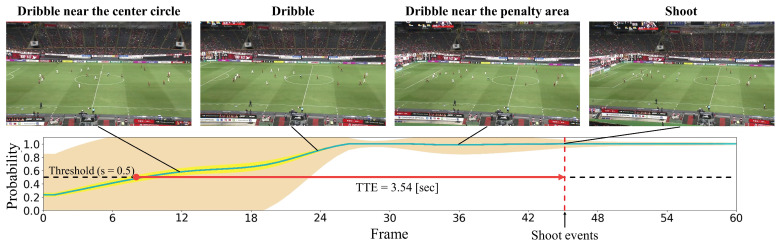
The qualitative prediction results of the proposed method for shoot event occurrence at a threshold of s=0.5. It displays an example of a positive sample with successful predictions. The proposed method predicts the probability of shoot event occurrence at each time step (indicated by the blue curve) and estimates both aleatoric uncertainty (shown in the orange region) and epistemic uncertainty (shown in the yellow region) for each time step.

**Figure 7:**
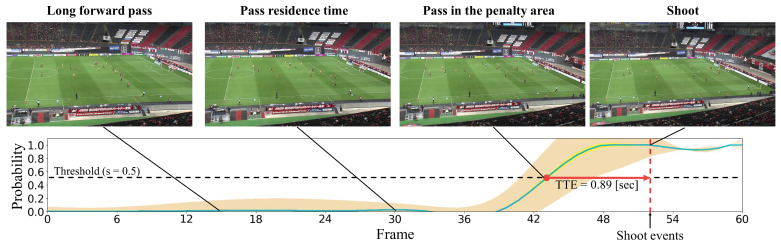
This figure illustrates an instance of accurate predictions for the positive sample. The details depicted are identical to those in Figure 6.

**Figure 8:**
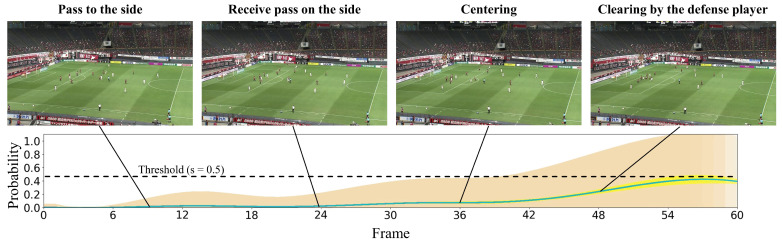
This figure presents an example of precise predictions for the negative sample. The specifics depicted are identical to those in Figure 6.

**Figure 9:**
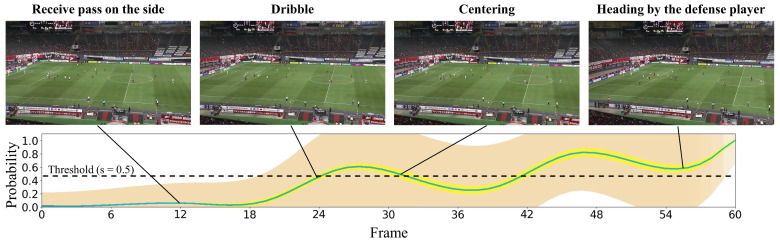
This figure demonstrates an instance of inaccurate predictions for the negative sample. The particulars shown are identical to those in Figure 6.

**Figure 10:**
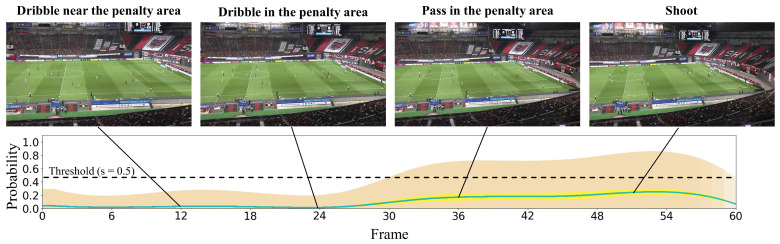
This figure displays an instance of erroneous predictions for the positive sample. The specifics depicted are identical to those in Figure 6.

In this subsection, we present the visualization of the shoot event prediction results obtained by the proposed method (refer to Figure 6, Figure 7, Figure 8, Figure 9 and Figure 10) and provide a qualitative assessment of the prediction performance. Figure 6, Figure 7, Figure 8, Figure 9 and Figure 10 depict the prediction examples of the proposed method. Specifically, Figure 6 and Figure 7 show successful predictions for positive samples, while Figure 8 is an example of successful negative sample predictions. Figure 9 and Figure 10 are false positive and false negative examples, respectively. To depict each outcome, a threshold value of 0.5 was established to determine whether the model predicted the occurrence of a shooting event in the future. Each example displays several frames of the video clip at the top of the figure. The bottom section of the figure portrays the probability of the shooting prediction (blue curve) and the corresponding aleatoric and epistemic uncertainties (orange and yellow regions, respectively) in the time series. Figure 6 is a brief counter-attack scene that may result in shooting events, and the probability of shooting prediction has reached the threshold value of 3.54 s before the time of the shooting event. In the case of Figure 7, a long forward pass was made, and the predicted probability rapidly increases around the successful pass, resulting in a TTA of 0.89 s. In the example of Figure 6, the model provides predictions with uncertainties during the increasing predicted probability phase. However, in the later phase, the model is relatively certain about its predictions, resulting in low uncertainties. In example of Figure 7, the uncertainty region demonstrates that the model provides predictions with uncertainties during the probability increase phase (when it is not yet known whether the long pass will be successful or not), in comparison to both the initial and later phases. In the case of Figure 8, which is an instance of a true negative depicting a centering scene, the probability does not attain the threshold value. Nonetheless, it suggests that there are significant uncertainties in the predicted outcomes. In the case of Figure 9, which is an instance of false positives and also a centering scene that does not culminate in a shooting event, the probability surpasses the threshold value with considerable uncertainty. In the case of Figure 9, an example of false positive, which is also a centering scene that does not lead to a shoot, the probability exceeds the threshold value with a large uncertainty. In soccer matches, it has been reported that the probability of a centering attack leading to a shoot is lower than that of a middle attack [60]. Therefore, in cases of uncertainty and false prediction results, this fact can be taken into consideration. In soccer matches, the probability that a centering attack leads to a shoot is lower than a middle attack [60], and thus it can be considered that it is confirmed in the case of uncertainty and false prediction results. In the case of Figure 10, which is a false negative example, several defensive players were positioned in front of the attacking players around the goal. It should be noted that the limitation of this study is that the ball carrier is unknown, thus the probability of shoot event occurrence may not have reached the threshold value, even though it occurred.

The proposed method demonstrates effectiveness in predicting the occurrence of shoot events, as evidenced by both qualitative and quantitative evaluations.

## 5. Conclusions

This paper proposes a shoot event prediction method that considers the spatial-temporal relationships between players in soccer videos using complete bipartite graphs. Specifically, the proposed method represents player relations as a complete bipartite graph using various visual features at each time step. The method then applies GCRNN to calculate latent features that consider the spatio-temporal relations between players. In the proposed method, the use of complete bipartite graphs enables the consideration of both players’ distance and team information, resulting in a more detailed representation of the match situation through the incorporation of player relations. The latent features obtained through GCRNN are then fed into the BNN, which uses them to make accurate predictions of the probability of a shooting event occurring. The BNN takes into account prediction uncertainty to produce reliable estimates. The experimental results using real soccer video clips demonstrate the effectiveness of the proposed method. Our shoot event prediction model is constructed for soccer videos. In soccer, players’ positions and their temporal changes are critical factors for scoring. Besides, to introduce shoot event prediction techniques into tactical coaching in soccer, the reliability of the prediction results should be uncovered. From these standpoints, the contributions of this paper are summarized as follows:

(1) We developed a new method for predicting shoot events in soccer videos, incorporating player-specific features and spatio-temporal relations with GCRNN.
(2) The proposed method provides a measure of prediction uncertainty by using BNN, which is intended to enhance the robustness of the predictions.

On the other hand, our shoot event prediction model still has the following limitations:

(1) Our model cannot consider the skill levels of each player that could change the development of the game. Annotations for each player are needed for introducing such skill levels, but manual annotation is not a realistic solution since it takes a lot of effort and time.
(2) Reflection of the ball position, which is the direct method for grasping the situations of the game, should be performed in our model. However, when using the scouting soccer videos for recognizing more players, the ball region is too small to be recognized by the object detector. The use of GPS Information is one possible solution, but it is difficult to implement in minor games.

As the applicability, we consider that our event prediction model can easily be applied into other sports. Here, soccer is the specific sport with the property that there is a large number of players and has a high degree of freedom for each player, and the event prediction task in soccer is more difficult than that of other sequentially sports, e.g., basketball, water polo, and rugby. Hence, our prediction model is likely to provide accurate event predictions for such sports, as it has been successful in soccer by capturing the more complex relation between players. Therefore, our model has the high applicability and great potential to provide well benefit for other sports.

## Figures and Tables

**Table 1:** Comparison of AP and F1-score among different video clip lengths for the proposed and comparison methods. Each video clip length is counted from the start of the video clip. The best performance at each video clip length is shown in bold.

	*t* = 10		*t* = 20		*t* = 30		*t* = 40		*t* = 50		*t* = 60	
	AP	F1-Score	AP	F1-Score	AP	F1-Score	AP	F1-Score	AP	F1-Score	AP	F1-Score
Ours	**0.960**	0.880	**0.966**	**0.909**	0.964	**0.923**	**0.964**	**0.923**	**0.970**	**0.921**	**0.963**	**0.914**
AS1 [37]	0.955	0.889	0.961	0.897	0.962	0.897	0.959	0.909	0.958	0.900	0.954	0.880
AS2	0.954	**0.892**	0.961	0.881	0.932	0.886	0.929	0.892	0.924	0.892	0.919	0.892
AS3	0.956	0.865	0.960	0.889	0.934	0.883	0.914	0.895	0.909	0.895	0.904	0.895
AS4	0.951	0.881	0.964	0.889	0.961	0.914	0.962	0.904	0.963	0.897	0.956	0.897
CM1 [53]	0.931	0.878	0.958	0.902	**0.966**	0.902	0.959	0.900	0.961	0.892	0.958	0.881
CM2 [55]	0.752	0.714	0.776	0.729	0.778	0.725	0.782	0.747	0.784	0.759	0.790	0.722

**Table 2:** AP, F1-score and mTTE at the time step $T_{o}$. Please, note that CM2 cannot handle varying video lengths, so it is denotes as ‘‘-’’. The best performance in terms of prediction accuracy is denoted in bold.

	AP	F1-Score	mTTE(s)
Ours	**0.967**	**0.914**	3.88
AS1 [37]	0.941	0.880	3.60
AS2	0.917	0.892	4.32
AS3	0.903	0.895	4.66
AS4	0.950	0.886	3.67
CM1 [53]	0.931	0.853	3.62
CM2 [55]	-	-	-

## Data Availability

Experimental data cannot be disclosed.
